# Low expression of RECQL is associated with poor prognosis in Chinese breast cancer patients

**DOI:** 10.1186/s12885-018-4585-1

**Published:** 2018-06-18

**Authors:** Huiying Xu, Ye Xu, Tao Ouyang, Jinfeng Li, Tianfeng Wang, Zhaoqing Fan, Tie Fan, Benyao Lin, Yuntao Xie

**Affiliations:** Key Laboratory of Carcinogenesis and Translational Research (Ministry of Education), Breast Center, Beijing Cancer Hospital & Institute, Peking University Cancer Hospital, Beijing, 100142 People’s Republic of China

**Keywords:** RECQL, Expression, Survival, Breast cancer

## Abstract

**Background:**

RECQL is a number of the RecQ DNA helicase family and plays an important role in maintaining genome stability. Although several studies have reported that RECQL mutations were correlated with the susceptibility to breast cancer, the effect on prognosis in breast cancer was not yet clarified. Here, we explored the association between RECQL expression level and survival in patients with breast cancer.

**Methods:**

In the first cohort, the RECQL mRNA expression level was evaluated in 774 primary breast cancer patients using a quantitative real-time PCR assay. Then, in the second independent cohort, the level of RECQL protein expression was detected in 322 patients with breast cancer using immunohistochemistry assay. Survival curves of patients with RECQL expression were compared using the Kaplan-Meier method with log-rank test.

**Results:**

In the first cohort of 774 breast cancer patients, the low expression level of RECQL mRNA was significantly correlated with aggressive clinicopathological characteristics, including the positive lymph node status (*P* = 0.026), HER2 overexpression (*P* < 0.001), ER negative status (*P* = 0.047) and high tumor grade (*P* = 0.041). Moreover, the low expression level of RECQL mRNA was significantly associated with poor distant recurrence-free survival (DRFS, unadjusted hazard ratio (HR): 2.77, 95% confidence interval (CI): 1.88–4.09, *P* < 0.001) and disease-specific survival (DSS, unadjusted HR: 3.10, 95% CI: 1.84–5.20,*P* < 0.001), and it remained an independent unfavorable factor for DRFS and DSS (DRFS: adjusted HR: 3.04, 95% CI: 1.89–4.87, *P* < 0.001; DSS: adjusted HR: 4.25, 95% CI: 2.12–8.46, *P* < 0.001). In the second cohort of 322 breast cancer patients, low expression of RECQL protein was also subject to poor survival in breast cancer, and it was an independent prognosis factor of poor DRFS by multivariate analysis (DRFS: adjusted HR: 2.12, 95% CI: 1.16–3.88, *P* = 0.015).

**Conclusions:**

Breast cancer patients with low RECQL expression had a worse survival. The expression level of RECQL may be a potential prognosis factor for breast cancer.

**Electronic supplementary material:**

The online version of this article (10.1186/s12885-018-4585-1) contains supplementary material, which is available to authorized users.

## Background

At present, breast cancer was one of the most prevalent cancers among women in the world, and seriously threatened the health of women [1]. Because of the biological heterogeneity of breast cancer, defining accurate prognostic and predictive biomarkers may be in favor of designing effective treatments for breast cancer patients [2].

RECQL is an ATP-dependent DNA helicase enzyme, which belongs to the family of RecQ helicase that plays an important role in mismatch repair, nucleotide excision repair and direct repair [3, 4]. RecQ helicase in human includes five members, namely RECQL, BLM, WRN, RECQL4 and RECQL5. Previous studies showed that the germline mutations in BLM, WRN and RECQL4 had high predisposition to cancer and premature aging [3, 4]. RECQL is the most abundant DNA helicase enzyme among RecQ helicase family and also has critical biological functions. RECQL has been shown to involve in DNA replication [5, 6], transcription [7], recombination [8] and repair [9, 10], restart stalled replication forks [11–13], and telomere maintenance [14]. Compared with wild-type mice, mice with deficient RECQL hadn’t any apparent phenotypic differences, but embryonic fibroblasts from RECQL-deficient mice exhibited many signs of genomic instability, such as aneuploidy, spontaneous, chromosomal breakage and frequent translocation events [15]. Besides, human RECQL-deficient cells turned out to be chromosomal instability, and hypersensitive to ionizing radiation [10, 15]. These results indicated that RECQL played an important role in maintaining genomic stability.

Previously, our lab and Cybulski et al. reported that RECQL gene mutations were correlated with high risk of breast cancer independently in Chinese [16] and Caucasian populations [17]. Kwong et al. also found six germline mutations in RECQL gene in 1110 patients with high risk breast cancer in Hong Kong [18]. At present, RECQL was demonstrated as a moderate breast cancer susceptibility gene and a tumor suppressor. Earlier studies revealed that single nucleotide polymorphisms of RECQL affected clinical prognosis of patients with pancreatic cancer [19, 20]. Nevertheless, few studies were performed on the specific effect of RECQL expression on breast cancer outcomes. In this study, we investigated the association between RECQL mRNA and protein expression and survival of breast cancer in two independent cohorts.

## Methods

### Study population

In the first cohort, the study samples were pretreatment core-needle biopsy specimens of 834 primary breast cancer patients (stage I-III) who were treated at the Breast Center, Peking University Cancer Hospital from 2004 to 2011. Of these, 60 specimens failed to assess the level of RECQL mRNA expression due to the poor quality of the RNA samples. Thus, a total of 774 breast cancer patients were analyzed in the first cohort. The patients’ age at diagnosis ranged from 25 to 93 years, whose median was 52 years. According to medical records, patients received either a mastectomy (*n* = 445) or a breast-conserving surgery (*n* = 294). The majority of patients received adjuvant therapy, including chemotherapy, endocrine therapy, or chemotherapy in combination with endocrine therapy. Thirty-five patients received adjuvant trastuzumab therapy (Table 1). The median follow-up of all 774 patients was 82 months (range 2 to 140 months). During follow-up period, 150 patients experienced distant recurrences or died of the disease.

**Table 1:** Association between RECQL mRNA Expression and Clinicopathologic Characteristics (*N* = 774)

Characteristic	No.	RECQL mRNA expression				P
		Low		High		
		No.	%	No.	%	
Total	774	387	50.0	387	50.0	
Age						
≤ 50 yr	331	168	43.4	163	42.1	0.72
> 50 yr	443	219	56.6	224	57.9	
Tumor size						
≤ 2 cm	298	142	36.7	156	40.3	0.30
> 2 cm	476	245	63.3	231	59.7	
Tumor grade						
I	186	85	22.7	101	26.6	0.041
II	443	215	57.5	228	60.2	
III	124	74	19.8	50	13.2	
Unknown	21	13		8		
Lymph node status						
Negative	510	239	65.5	271	73.0	0.026
Positive	226	126	34.5	100	27.0	
Unknown	38	22		16		
ER status						
Negative	223	124	32.1	99	25.6	0.047
Positive	549	262	67.9	287	74.4	
Unknown	2	1		1		
PR status						
Negative	302	160	42.0	142	37.1	0.16
Positive	462	221	58.0	241	62.9	
Unknown	10	6		4		
HER2 status						
Negative	556	254	66.0	302	78.2	< 0.001
Positive	215	131	34.0	84	21.8	
Unknown	3	2		1		
Ki-67						
High	445	233	62.3	212	55.9	0.08
Low	308	141	37.7	167	44.1	
Unknown	21	13		8		
Subtype						
Luminal A	173	76	19.8	97	25.1	0.003
Luminal B(HER2-)	268	119	30.1	149	38.6	
Luminal B(HER2+)	123	78	20.3	45	11.7	
HER2+	92	53	13.8	39	10.1	
TN	114	58	15.1	56	14.5	
Unknown	4	3		1		
Adjuvant Therapy						
C	157	83	21.4	74	19.1	0.011
E	281	121	31.3	160	41.3	
C + E	245	127	32.8	118	30.5	
No therapy	91	56	14.5	35	9.0	
Trastuzumab use						
No	739	372	96.1	367	94.8	0.39
Yes	35	15	3.9	20	5.2	
Surgery type						
BCS	294	137	37.4	157	42.1	0.20
Mastectomy	445	229	62.6	216	57.9	
Unknown	35	21		14		

Abbreviations: ER, estrogen receptor; PR, progesterone receptor; HER2, human epidermal growth factor receptor-2; TN, triple negative; C, chemotherapy; E, endocrinotherapy; C + E, chemotherapy and endocrinotherapy; BCS, breast-conserving surgery

Comments: luminal A: ER+ or PR ≥ 20%, HER2-, Ki-67 < 14%; luminal B (HER2-): ER+ and HER2-, Ki-67 ≥ 14% or PR−/< 20%, luminal B (HER2+): ER+ and HER2+; HER2(+): ER- and PR-, HER2+; TN: ER- and PR-, HER2-

To further clarify the conformance to the results of the first cohort, we analyzed another independent cohort of patients in this study (cohort 2). In cohort 2, paraffin blocks of tumor tissues were available for 358 primary breast cancer patients (stage I-III) who were treated at Breast Center, Peking University Cancer Hospital from January 2001 to June 2002. Among these, 18 patients lost the follow-up, and 18 tumor specimens were failed to assess RECQL staining because of tissue loss during the experiment. Finally, 322 patients were analyzed in cohort 2. The mean patients’ age was 50 years (range 25 to 88 years), and the median follow-up of these patients was 98 months (range 2 to 129 months). All patients received modified radical mastectomy surgery. The majority of patients (90.4%, 291/322) received adjuvant therapy after surgery. None of them received adjuvant trastuzumab therapy (Table 2).

**Table 2:** Association between RECQL Protein Expression and Clinicopathologic Characteristic (N = 322)

Characteristic	No.	RECQL protein expression				
		Low		High		p
		No.	%	No.	%	
Total	322	133	41.3	189	58.7	
Age						
≤ 50 yr	153	74	54.4	82	43.4	0.07
> 50 yr	169	62	45.6	107	56.6	
Tumor size						
≤ 2 cm	112	48	45.7	64	43.5	0.73
> 2 cm	140	57	54.3	83	56.5	
Unknown	70	28		42		
Lymph node status						
Negative	168	75	59.1	93	51.1	0.17
Positive	141	52	40.9	89	48.9	
Unknown	13	6		7		
ER status						
Negative	100	44	43.6	56	35.9	0.22
Positive	157	57	56.4	100	64.1	
Unknown	65	30		33		
PR status						
Negative	101	33	33.3	68	43.3	0.11
Positive	155	66	66.7	89	56.7	
Unknown	66	34		32		
HER2 status						
Negative	233	98	78.4	135	78.0	0.94
Positive	65	27	21.6	38	22.0	
Unknown	24	8		16		
TN						
Yes	29	7	7.8	22	14.8	0.19
No	210	83	92.2	127	85.2	
Unknown	83	43		40		
Adjuvant Therapy						
C	124	58	43.6	66	34.9	0.463
E	20	7	5.3	13	6.9	
C + E	147	56	42.1	91	48.1	
No therapy	31	12	9.0	19	10.1	

Abbreviations: ER, estrogen receptor; PR, progesterone receptor; HER2, human epidermal growth factor receptor-2; TN, triple negative; C, chemotherapy; E, endocrinotherapy; C + E, chemotherapy and endocrinotherapy

The tumor size, grade, and stage were classified as same as our previous study [21]. This study was approved by the Research and Ethical Committee of Peking University Cancer Hospital.

### Pathology

These breast cancer tissues were obtained by the core-needle biopsy, estrogen receptor (ER), progesterone receptor (PR), and human epidermal growth factor receptor 2 (HER2) were determined by an immunohistochemical (IHC) assay as described previously [21]. In the present study, ER or PR was considered positive when it had ≥1% positive nuclear staining tumor cells. HER2 was deemed to be positive when its immunohistochemical score was 3+ or the fluorescence in situ hybridization assay showed HER2 gene amplification [22].

### RECQL mRNA expression analysis by real-time quantitative PCR

In cohort 1, breast tumor RNA extracting and then transcribing RNA to cDNA were done according to the manufacturer’s instructions as described previously [23].

Real-time PCR of the RECQL gene was performed as described previously [23]. The primers for target gene RECQL were as follow: 5’- ACAAAATGTGCGATAACTGCTG-3’ and 5’-GCACCCTTTCCCATCCAAGA-3’. The sequences of the primers for endogenous control β-actin were 5’-GACAGGATGCAGAAGGAGATCACT-3’ and 5’-GTCAAGAAAGGGTGTAACGCAACT-3’. The PCR conditions were: 95 °C for 5 min followed by 40 cycles of 95 °C for 5 s, 60 °C for 30 s and final stage was followed by a melting curve from 60 °C to 95 °C. Each sample was assayed in triplicate with RNase-free water as negative control. Relative RECQL mRNA expression quantifications were calculated according to the formula 2 ^–ΔΔCt^. In the experiment, β-actin was an endogenous control and 293 cell line RNA control was a calibrator in each plate. The result showed the mean amplification efficiency of RECQL is 97% and β-actin’s is 94%. A melting curve analysis was completed to confirm the specificity of amplification in the each run.

Median of the relative gene expression values was selected as cutoff value to estimate the level of RECQL expression. Patients whose relative gene expression values were above the cutoff value were considered to be high expression of RECQL mRNA, while the rest of patients were low expression. Therefore, the 774 patients were divided into the high mRNA expression group (*n* = 387) and the low mRNA expression group (*n* = 387).

### RECQL protein expression analysis by immunohistochemical assay

In cohort 2, the tumor section (4 μm thick) was used for immunohistochemical staining. At first, tissue slides were dewaxed twice with xylene, then rehydrated through a graded alcohol series and immersed for 20 min in a 3% hydrogen peroxide buffer. After ddH_2_O rinsing, the tissue slides were putted into a box filled with EDTA buffer (pH 9.0) and then putted the box into a water bath at 95 °C for 25 min to retrieve antigen. The tissue slides were washed with 1× PBS for 5 min, blocked with normal goat serum for 30 min, and then incubated overnight at 4 °C in a humidified chamber with the primary anti-RECQL antibody (Bethyl Laboratories, catalog No.A300-450A) at a dilution of 1:2000. The sections were rinsed three times with 1× PBS and incubated with secondary antibody (ZSGB-BIO, catalog No.PV-6000) at room temperature for 60 min. The sections were DAB development and counterstained with hematoxylin. Negative controls were concluded each run to ensure that all the staining was specific.

The staining intensity in the nuclear and staining percentage of tumor cells were both evaluated: no staining was given a score 0; a faint, moderate or strong staining was scored as 1, 2or 3, respectively. The staining percentage of tumor cells was estimated (0–100%). When the value calculated by multiplication of the staining intensity and staining percentage was more than 100%, the tumor specimen was regarded as RECQL protein expression high. Each immunostained slide was evaluated by two blinded independent pathologists. Re-examinations were conducted when evaluations were discrepancies.

### Statistical analysis

The Pearson’s χ2 test was used to analyze the associations between the expression level of RECQL and clinicopathological features. For the survival analyses, distant recurrence-free survival (DRFS) was defined as the time from the date of diagnosis to the first distant recurrence or the occurrence of breast cancer related-death without a recorded relapse. Disease-specific survival (DSS) was defined as the time from date of diagnosis to the occurrence of death where breast cancer was the primary or underlying cause of death. Survival curve was performed using the Kaplan-Meier method with the log-rank test. A Cox regression model was performed in multivariate analysis. A *P* value < 0.05 with a two-sided was considered significant statistically. The SPSS Statistics 20.0 software (Chicago, USA) was used to analyze all data in the study.

## Results

### Clinicopathologic characteristics

In cohort 1, RECQL mRNA expression was measured successfully in 774 patients. Totally, 387 patients (50%) exhibited high level of RECQL mRNA expression and the remaining 50% of tumors exhibited low expression of RECQL mRNA based on the median as cut-off value. According to the clinicopathological characteristics showed in Table 1, patients with low RECQL mRNA expression tend to be lymph node-positive (34.5% vs. 27.0%, *P* = 0.026), tumor grade III (19.8% vs.13.2%, *P* = 0.041), HER2-positive (34.0% vs. 21.8%, *P* < 0.001), ER-negative (32.1% vs. 25.6%,*P* = 0.047), and Luminal B (HER2+) subtype (*P* = 0.003) (Table 1). However, the expression level of RECQL mRNA was not associated with diagnosis age, tumor size, and PR status (Table 1).

**Fig. 1:**
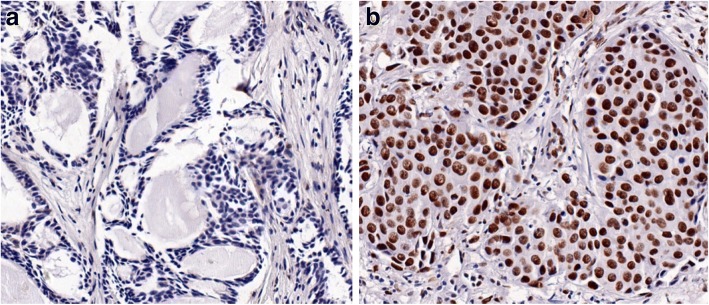
Immunohistochemical staining for RECQL expression in breast cancers. RECQL showed **a** negative staining and **b** positive staining (magnification × 200)

In the cohort 2, RECQL is mainly expressed in the cell nucleus (Fig. 1). Among 322 patients, 189 (58.7%) patients exhibited high level of RECQL protein expression and the remaining 133 (41.3%) patients exhibited low expression of RECQL protein expression. In this cohort, there was no correlation between RECQL protein expression and tumor size, age of diagnosis, ER, PR and HER2 status, and lymph nodes status (Table 2).

### RECQL mRNA expression associated with survival in cohort 1

In cohort 1, 774 patients were qualified for research and being analyzed. The median follow-up was 82 months (range 2 to 140 months) in cohort 1. The 10-year DRFS and DSS rates in 774 patients were 81.7% (95% confidence interval (CI): 78.6–84.8%), and 87.6% (95% CI: 84.5–90.7%), respectively.

**Fig. 2:**
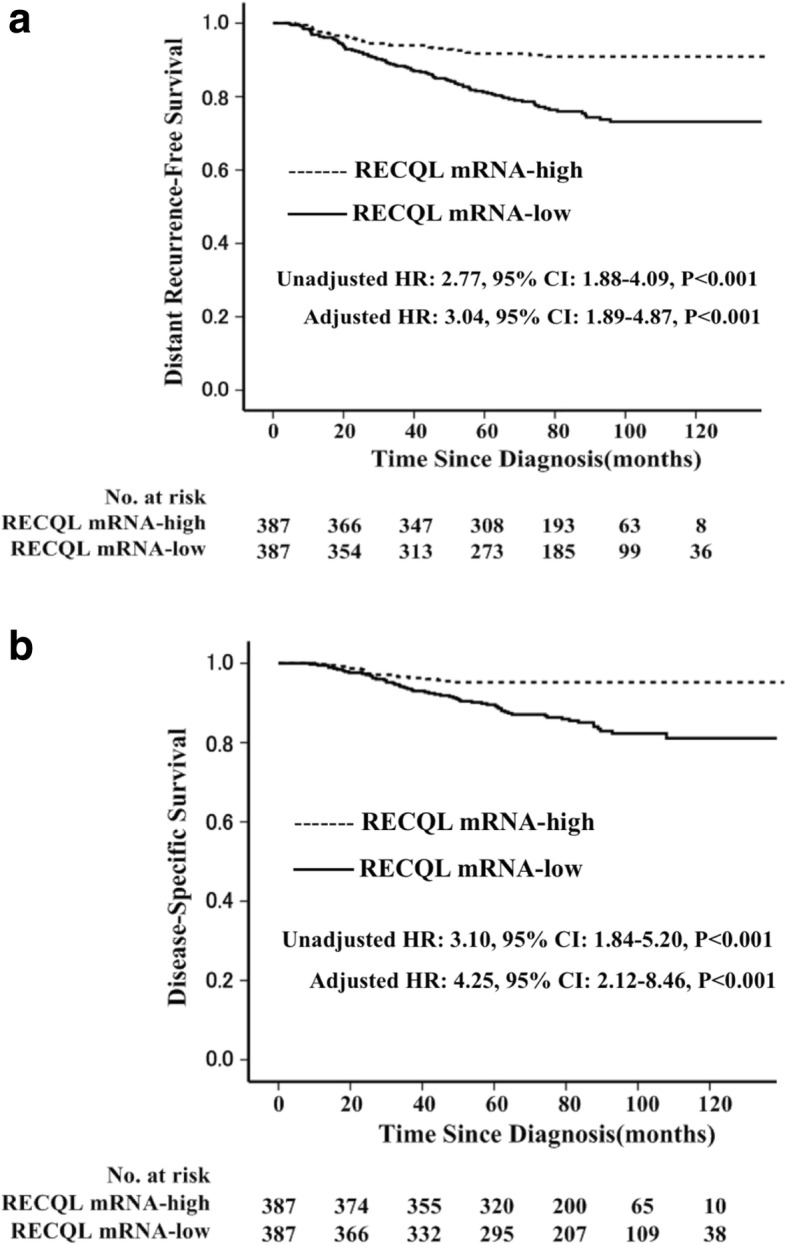
Comparison of the prognosis between RECQL mRNA expression high and low patients in the first cohort (*n* = 774) using Kaplan–Meier method. There was significant difference in distant recurrence-free survival (**a**) and disease-specific survival (**b**) between RECQL mRNA expression high and low patients

RECQL mRNA expression was significantly associated with survival in cohort 1 with 774 patients. Patients (*n* = 387) with low expression of RECQL mRNA had a significantly worse DRFS and DSS than did those with high level (DRFS, unadjusted HR: 2.77, 95% CI: 1.88–4.09, *P* < 0.001; DSS: unadjusted HR: 3.10, 95% CI: 1.84–5.20, *P* < 0.001) (Fig. 2a-b). Moreover, multivariate analysis revealed that low level expression of RECQL mRNA was an independent unfavorable factor for DRFS and DSS (DRFS: adjusted HR: 3.04, 95% CI: 1.89–4.87, *P* < 0.001; DSS: adjusted HR: 4.25, 95% CI: 2.12–8.46, *P* < 0.001) in these 774 patients after adjustment for tumor size, diagnosis age, ER status, PR status, HER2 status, histological grade, lymph node status and adjuvant therapy (Table 3). Lymph node positive was also independent unfavorable factor for DRFS (*P* < 0.001) and DSS (*P* < 0.001) (Table 3).

**Table 3:** Multivariate Analyses of Survival in the First Study Population (*N* = 774)

Variable	DRFS			DSS		
	HR	95% CI	P	HR	95% CI	P
Age						
≤ 50 yr. vs. > 50 yr	0.84	0.56–1.26	0.40	0.63	0.36–1.10	0.10
ER status						
Negative vs. Positive	1.93	0.98–3.79	0.06	1.51	0.62–3.72	0.37
PR status						
Negative vs. Positive	1.32	0.77–2.25	0.31	2.13	1.09–4.15	0.027
HER2 status						
Positive vs. Negative	0.88	0.56–1.39	0.59	0.75	0.42–1.36	0.34
Tumor grade						
III vs. I/II	1.18	0.70–1.99	0.53	1.47	0.77–2.80	0.24
Tumor size						
> 2 cm vs. ≤2 cm	1.52	0.97–2.38	0.07	2.26	1.17–4.40	0.017
Adjuvant chemotherapy						
C vs. no therapy	0.91	0.42–1.97	0.81	1.63	0.55–4.85	0.38
E vs. no therapy	1.14	0.47–2.80	0.77	1.71	0.47–6.25	0.42
C + E vs. no therapy	1.63	0.69–3.85	0.26	2.00	0.56–7.16	0.29
Lymph node status						
Positive vs. Negative	3.56	2.35–5.38	< 0.001	4.09	2.35–7.10	< 0.001
RECQL mRNA expression						
Low vs. High	3.04	1.89–4.87	< 0.001	4.25	2.12–8.46	< 0.001

Abbreviations: DRFS, distant recurrence-free survival; DSS, disease-specific survival; HR, hazard ratio; CI, confidence interval; ER, estrogen receptor; PR, progesterone receptor; HER2, human epidermal growth factor receptor-2;C,chemotherapy; E, endocrinotherapy; C + E, chemotherapy and endocrinotherapy

These 774 patients with breast cancer were further divided into five different subtypes according to ER\PR\HER2\Ki-67 status, and then survival analyses were performed in each subtype. The results showed that patients with RECQL mRNA low expression had worse survival compared with those with high expression in the luminal A, luminal B (HER2-), luminal B (HER2+) and the triple negative subtype (Additional file 1: Figure S1 A-D). However, in the HER2+ subtype, there was no significant difference between patients expressed high RECQL mRNA and low (Additional file 1: Figure S1E).

### RECQL protein expression associated with survival in cohort 2

**Fig. 3:**
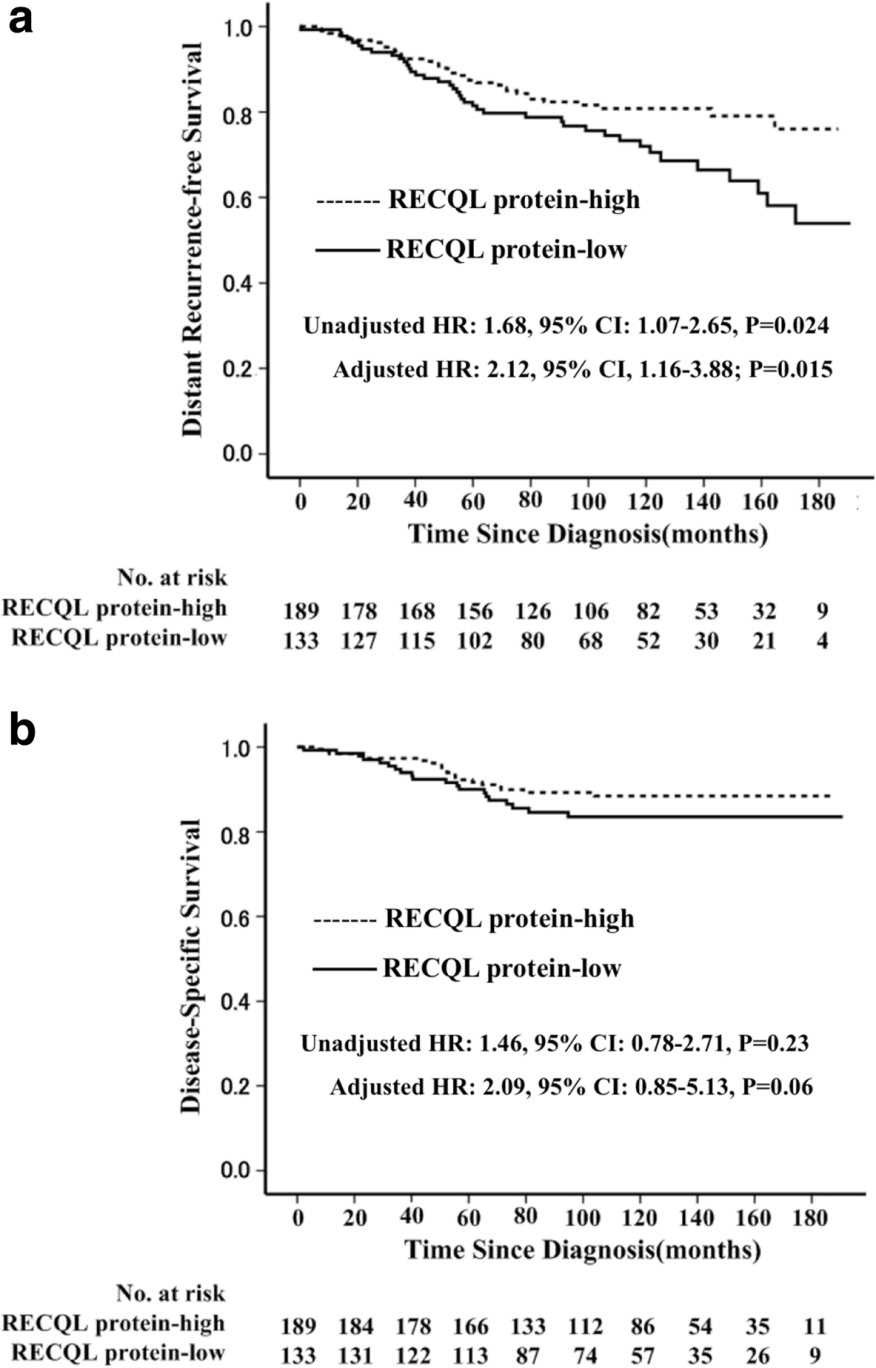
Comparison of the prognosis between RECQL protein expression high and low patients in the second cohort (*n* = 332) using Kaplan–Meier method. There was significant difference in distant recurrence-free survival (**a**) and disease-specific survival (**b**) between RECQL protein expression high and low patients

The results from the cohort 1 showed that RECQL mRNA expression level was significantly associated with survival of breast cancer patients. To verify the finding in protein level, another independent cohort of 322 breast cancer patients (I-III stage) was included for analysis. The median follow-up was 98 months (range 2 to 129 months) in cohort 2. The 10-year DRFS and DSS rates in the entire study population (*n* = 322) were 77.3% (95% CI: 72.4–82.2%), and 86.4% (95% CI: 82.5–90.3%), respectively. Compared with patients (*n* = 189) with high RECQL protein expression in tumors, patients (*n* = 133) with the low level of RECQL protein expression in tumors had worse DRFS (10-DRFS: 81.5%vs. 87.5%, *P* = 0.024), but no significant difference in DSS (10-DSS: 90.1% vs. 92.3%, *P* = 0.23) in univariate analysis (Fig. 3a-b). Moreover, a multivariable analysis revealed that the low level of RECQL protein expression was an independent unfavorable factor for DRFS (adjusted HR: 2.12, 95% CI, 1.16–3.88; *P* = 0.015) in these 322 patients after adjustment for age of diagnosis, lymph node status, PR status, ER status, HER2 status, tumor size and adjuvant therapy (Table 4).

**Table 4:** Multivariate Analyses of Survival in the Second Study Population (N = 322)

Variable	DDFS			DSS		
	HR	95% CI	P	HR	95% CI	P
Age						
> 50 yr. vs. ≤50 yr	0.56	0.29–1.07	0.08	0.34	0.13–0.92	0.033
ER status						
Negative vs. Positive	1.01	0.49–2.06	0.99	0.58	0.19–1.75	0.33
PR status						
Negative vs. Positive	0.82	0.39–1.72	0.60	0.86	0.30–2.50	0.79
HER2 status						
Positive vs. Negative	1.72	0.80–3.73	0.17	4.23	1.60–11.46	0.004
Tumor size						
> 2 cm vs. ≤2 cm	0.76	0.40–1.42	0.39	1.01	0.41–2.50	0.98
Lymph node status						
Positive vs. Negative	2.68	1.43–5.03	0.002	3.68	1.39–9.75	0.009
Adjuvant chemotherapy						
C vs. no therapy	1.71	0.38–7.79	0.49	1.46	0.29–7.29	0.64
E vs. no therapy	2.53	0.44–14.62	0.30	1.67	0.20–13.69	0.63
C + E vs.no therapy	2.11	0.47–9.47	0.33	0.92	0.17–5.01	0.63
RECQL protein expression						
Low vs. High	2.12	1.16–3.88	0.015	2.09	0.85–5.13	0.06

Abbreviations: RFS, recurrence-free survival; DRFS, distant recurrence-free survival; DSS, disease-specific survival; HR, hazard ratio; CI, confidence interval; ER, estrogen receptor; PR, progesterone receptor; HER2, human epidermal growth factor receptor-2;C,chemotherapy; E, endocrinotherapy; C + E, chemotherapy and endocrinotherapy

## Discussion

In this study, we investigated the association between RECQL expression level and survival in patients with breast cancer. We found that breast cancer patients with low RECQL expression had a worse survival than those with high level. This finding was replicated in mRNA and protein level in two independent cohorts, respectively. In cohort 1 (*N* = 774), patients with low expression level of RECQL mRNA had significantly poorer DRFS and DSS than those with the high level of RECQL expression. In order to further validate this finding in protein level, we analyzed RECQL protein expression in an independent cohort (*N* = 322). In the second cohort, patients with low protein level of RECQL also had lower DRFS than did patients with high level.

The RECQ DNA helicase family has five members, namely WRN, BLM, RECQL, RECQL4, and RECQL5. The biological functions of RECQ DNA helicases are inconsistence between members and they have different expression levels in the same tumor [24]. RECQL is the smallest and most abundant human RecQ helicase, and plays an important role in DNA repair and maintaining replication fork progression [25]. One study showed that RECQL deficiency could lead to chromosomal instability [15]. Recent studies reported that RECQL was a moderate breast cancer susceptibility gene [16–18, 26]. About prognostic studies, there was only one study evaluating the correlation of RECQL expression and survival in breast cancer from England population. Aroraet et al. reported that the low level of RECQL expression was associated with poorer survival than did those with high level expression [27]. By extending their findings, we could demonstrate that RECQL expression was strongly associated with worse DRFS and DSS in Chinese women with breast cancer. Moreover, RECQL expression remained an independent unfavorable factor after adjusting age of diagnosis, ER status, PR status, HER2 status, grade, tumor size, lymph node and adjuvant therapy. In addition, low RECQL expression was also associated with tumor grade III, lymph node-positive, HER2-positive, ER-negative, and tended to Luminal B (HER2+) subtype. These indicated that RECQL may associate with malignant phenotype in breast cancer.

However, some previous studies reported that patients with high level of RECQL expression tend to have a poorer prognosis than patients with low expression in multiple myeloma or epithelial ovarian cancer [28, 29]. Those findings were inconsistent with the results of breast cancer in this study. RECQL is the most expressive member of RecQ helicases and involves in DNA replication [5, 6], DNA repair and stability. RECQL has different expression level in different tumors. Highly proliferative cancer cells would probably need more RECQL for DNA replication and survival. RECQL was overexpressed in multiple myeloma and ovarian cells [28, 29], but RECQL expression was similar in breast cancer cell (MCF 7) relative to normal cells [30, 31]. Therefore, for these highly proliferative cancers, high level of RECQL expression is a bad secondary phenotype.

Some in vitro functional studies showed that RECQL-deficient tumor cells were more sensitive to DNA-toxic drugs [32, 33]. Another study showed that RECQL-overexpression in myeloma cells were resistant to melphalan and bortezomib, whereas silencing RECQL expression can make cells more sensitive to these two drugs [28]. We also analyzed 487 breast cancer patients who received neoadjuvant chemotherapy from the cohort 1. Among them, 18.9% (92/487) of patients achieved pathological complete remission (pCR). The result showed that there was no significant association between RECQL mRNA expression and the efficacy of neoadjuvant chemotherapy in breast cancers (data not shown).

The underlying mechanism of RECQL expression affect breast cancer prognosis was not yet clear. Several studies have showed that RECQL played an important role in maintaining genomic stability [3, 4]. When the expression of RECQL is insufficient, RECQL maybe not play its normal role in maintaining genomic stability, which makes the tumor cells more likely to undergo malignant transformation, and ultimately lead to poor prognosis of breast cancer. Further functional studies are needed to clarify the underlying mechanism.

There are also some limitations in this study. RECQL mRNA and protein were not assayed in the same samples, so the consistency of the mRNA and protein expression level can’t be evaluated.

## Conclusions

In summary, in this study we found that the low RECQL expression is strongly associated with poor prognosis in breast cancer. RECQL expression may be a useful marker in estimating the prognosis of breast cancer patients. Nevertheless, further functional and independent studies are warranted to confirm our findings.

## Additional file

Additional file 1: **Figure S1.** Comparison of the prognosis between RECQL mRNA expression high and low patients in (A) luminal A, (B) luminal B (HER2-), (C) luminal B (HER2+), (D) triple negative, and (E)HER2(+)subtype using Kaplan–Meier method. Comments: luminal A: ER+ or PR ≥ 20%, HER2-, Ki-67 < 14%; luminal B (HER2-): ER+ and HER2-, Ki-67 ≥ 14% or PR−/< 20%, luminal B (HER2+): ER+ and HER2+;HER2(+): ER- and PR-, HER2+; TN (triple negative): ER- and PR-, HER2-. (DOCX 502 kb)

## Abbreviations

CI: Confidence interval
DRFS: Distant recurrence-free survival
DSS: Disease-specific survival
ER: Estrogen receptor
HER2: Human epidermal growth factor receptor-2
HR: Hazard ratio
IHC: Immunohistochemical
PCR: Polymerase chain reaction
PR: Progesterone receptor
TNM: Tumor node metastasis
