# Supplementary figures and images for: Low expression of RECQL is associated with poor prognosis in Chinese breast cancer patients
Source: BMC Cancer. 2018 Jun 18;18:662. doi: 10.1186/s12885-018-4585-1 (PMC6007067; doi:10.1186/s12885-018-4585-1)

**Figure S1**

**A.**

**
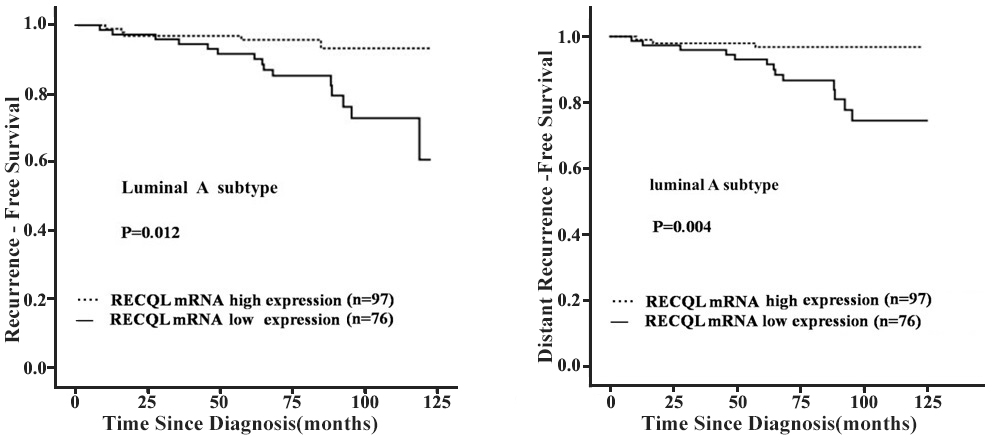
**

**B.**

**
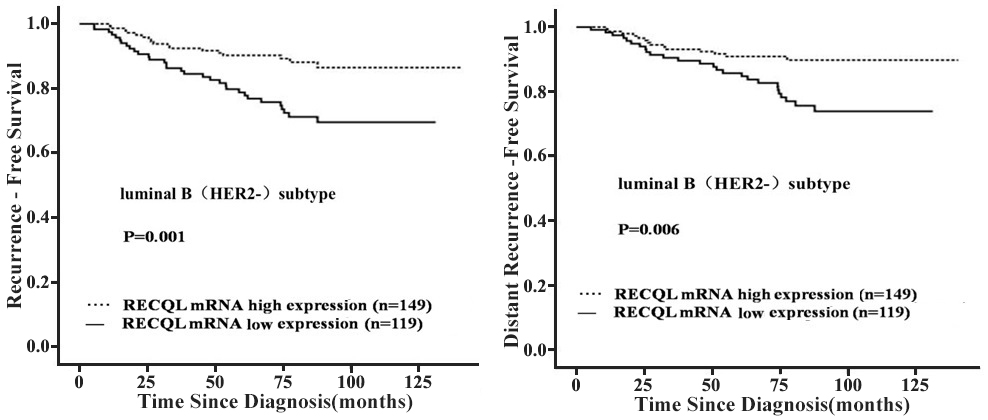
**

**C.**

**
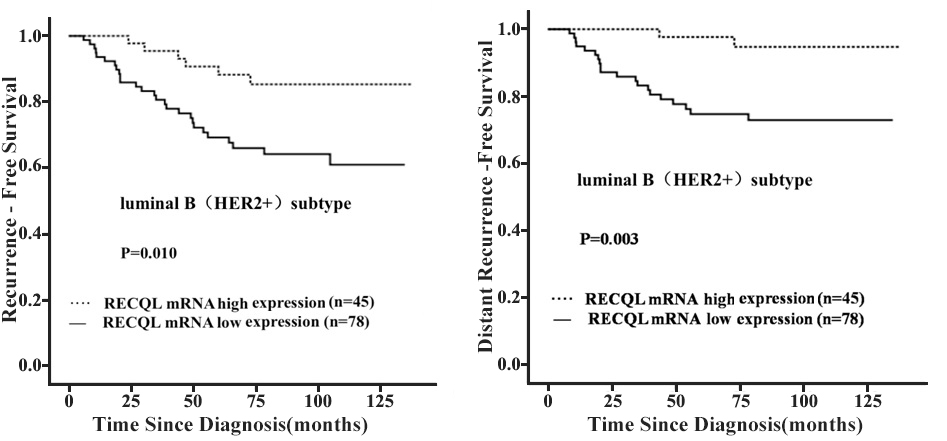
**

**D**

**
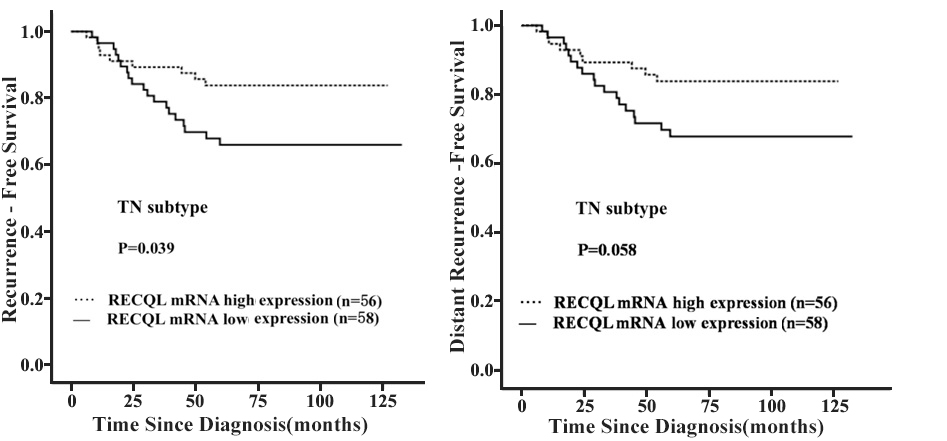
**

**E.**

**
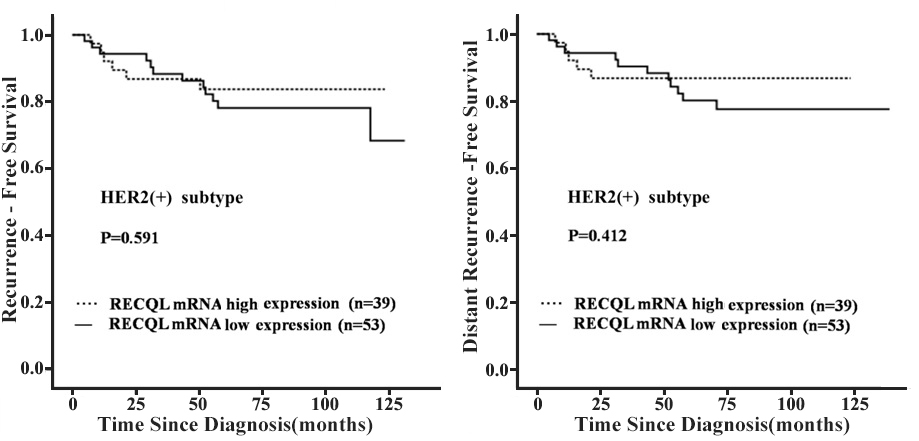
**

Supplement: Supplementary file 1 — Figure S1. Comparison of the prognosis between RECQL mRNA expression high and low patients in (A) luminal A, (B) luminal B (HER2-), (C) luminal B (HER2+), (D) triple negative, and (E)HER2(+)subtype using Kaplan–Meier method. Comments: luminal A: ER+ or PR ≥ 20%, HER2-, Ki-67 < 14%; luminal B (HER2-): ER+ and HER2-, Ki-67 ≥ 14% or PR−/< 20%, luminal B (HER2+): ER+ and HER2+;HER2(+): ER- and PR-, HER2+; TN (triple negative): ER- and PR-, HER2-. (DOCX 502 kb) [file 12885_2018_4585_MOESM1_ESM.docx]
